# Illumination matters part IV: blackout and whiteout in flexible ureteroscopy - first report on a phenomenon observed by PEARLS

**DOI:** 10.1007/s00345-024-04988-1

**Published:** 2024-05-05

**Authors:** Jia-Lun Kwok, Vincent De Coninck, Frédéric Panthier, Hatem Kamkoum, Felipe Pauchard, Anil Shrestha, Vineet Gauhar, Jan Brachlow, Florian Alexander Schmid, Cédric Poyet, Daniel Eberli, Olivier Traxer, Etienne Xavier Keller

**Affiliations:** 1 Department of Urology, University Hospital Zurich, Frauenklinikstrasse 10, CH-8091 Zurich, Switzerland; 2 Department of Urology, Tan Tock Seng Hospital, Singapore, Singapore; 3 Progressive Endourological Association for Research and Leading Solutions (PEARLS), Paris, France; 4 Endourology & Urolithiasis Working Group, Young Academic Urologists (YAU), Arnhem, The Netherlands; 5 Department of Urology, AZ Klina, Brasschaat, Belgium; 6 GRC N°20, Groupe de Recherche Clinique sur la Lithiase Urinaire, Hôpital Tenon, Sorbonne Université, 75020 Paris, France; 7 Hamad Medical Corporation, Doha, Qatar; 8 Urology Department, Hospital Naval Almirante Nef, 2520000, Viña del Mar, Chile; 9 Department of Urology, National Academy of Medical Sciences, Bir Hospital and B&B Hospital, Gwarko Lalitpur, Nepal; 10 Department of Urology, Ng Teng Fong General Hospital, Singapore, Singapore; 11 Zentrum Für Urologie Winterthur, Winterthur, Switzerland

**Keywords:** Flexible ureteroscopy, Blackout, Whiteout, Stone disease, Image

## Abstract

**Purpose:**

To date, no study has evaluated effects of varying brightness settings on image quality from flexible ureteroscopes submerged in saline. The aim was to evaluate blackout and whiteout occurrences in an in-vitro kidney calyx model.

**Material and methods:**

We evaluated a series of contemporary flexible ureteroscopes including the Storz Flex-Xc and Flex-X2s, Olympus V3 and P7, Pusen 7.5F and 9.2F, as well as OTU WiScope using a 3D-printed enclosed pink in-vitro kidney calyx model submerged in saline. Endoscopic images were captured with ureteroscope tip placed at 5 mm,10 mm and 20 mm distances. The complete range of brightness settings and video capture modes were evaluated for each scope. Distribution of brightness on a grayscale histogram of images was analyzed (scale range 0 to 255). Blackout and whiteout were defined as median histogram ranges from 0 to 35 and 220 to 255, respectively (monitor image too dark or too bright for the human eye, respectively).

**Results:**

Blackout occurred with the P7, Pusen 7.5F, 9.2F and WiScope at all distances, and V3 at 20 mm - with lowest brightness settings. Whiteout occurred with Flex-X2s, V3 and P7 at 5 mm and 10 mm, as well as with V3 and P7 at 20 mm - mostly with highest brightness settings. The Flex-Xc had neither blackout nor whiteout at all settings and distances.

**Conclusion:**

Blackout or whiteout of images is an undesirable property that was found for several scopes, possibly impacting diagnostic and therapeutic purposes during ureteroscopy. These observations form a guide to impact a urologist’s choice of instruments and settings.

## Introduction

Ureteroscopy treatment has been increasing in various countries worldwide over the last 2 decades [1–3]. Quality of optics is an important part of evaluation of flexible ureteroscopes [4]. Imaging properties of flexible ureteroscopes have been extensively evaluated - mostly in air [4], and recently in saline [5]. Visibility has been prior evaluated [4], but no study to date has evaluated the effects of varying brightness settings on image quality from ureteroscopes submerged in saline - the medium used for most endoscopic interventions [6–23]. The authors have noticed that at low brightness settings for some scopes, there is blackout - where the endoscopic image is too dark to visualize the endoscopic field well. At higher brightness settings, the authors have noticed whiteout - where the endoscopic image is too bright to visualize the endoscopic field well. This phenomenon has not been objectively evaluated and reported before. The aim of the present study was to evaluate blackout and whiteout occurrences in an *in-vitro* kidney calyx model with various brightness settings and brightness modes.

## Material and methods

A range of ureteroscopes accessible at University Hospital Zurich were evaluated, including the Flex-Xc and Flex-X2s (Karl Storz SE & Co. KG, Tuttlingen, Germany), the URF-P7 and URF-V3 (Olympus, Center Valley, PA, USA), and single-use scopes Uscope 7.5F PU3033A, Uscope 9.2F PU3022A (Zhuhai Pusen Medical Technology Co. Ltd. Guangdong, China) and WiScope (OTU Medical Inc, CA, USA). To simulate routine clinical conditions, the single-use scopes (Pusen 7.5F, Pusen 9.2F and OTU WiScope) were unused from sealed sterile packages. The reusable scopes (Storz and Olympus scopes) had all been processed and decontaminated post clinical use, with no record of prior usage numbers.

For the Storz Flex-X2s, the Power LED 175 light source (unit usage < 100 h) was utilized with a corresponding 230 cm and 3.5 mm fiberoptic cable, together with the IMAGE1 S HX-P HDTV 1-Chip pendular camera (Karl Storz SE & Co. KG, Tuttlingen, Germany). With the Olympus URF-P7 and URF-V3, the VISERA elite CLV-S190 light source (Xenon short-arc lamp used < 100 h) was paired with a WA03310A 300 cm and 4.3 mm fiberoptic light cable, and the CH S190 08 LB camera head (Olympus, Center Valley, PA, USA) were used. Fiberoptic cables used were entirely new.

**Fig. 1:**
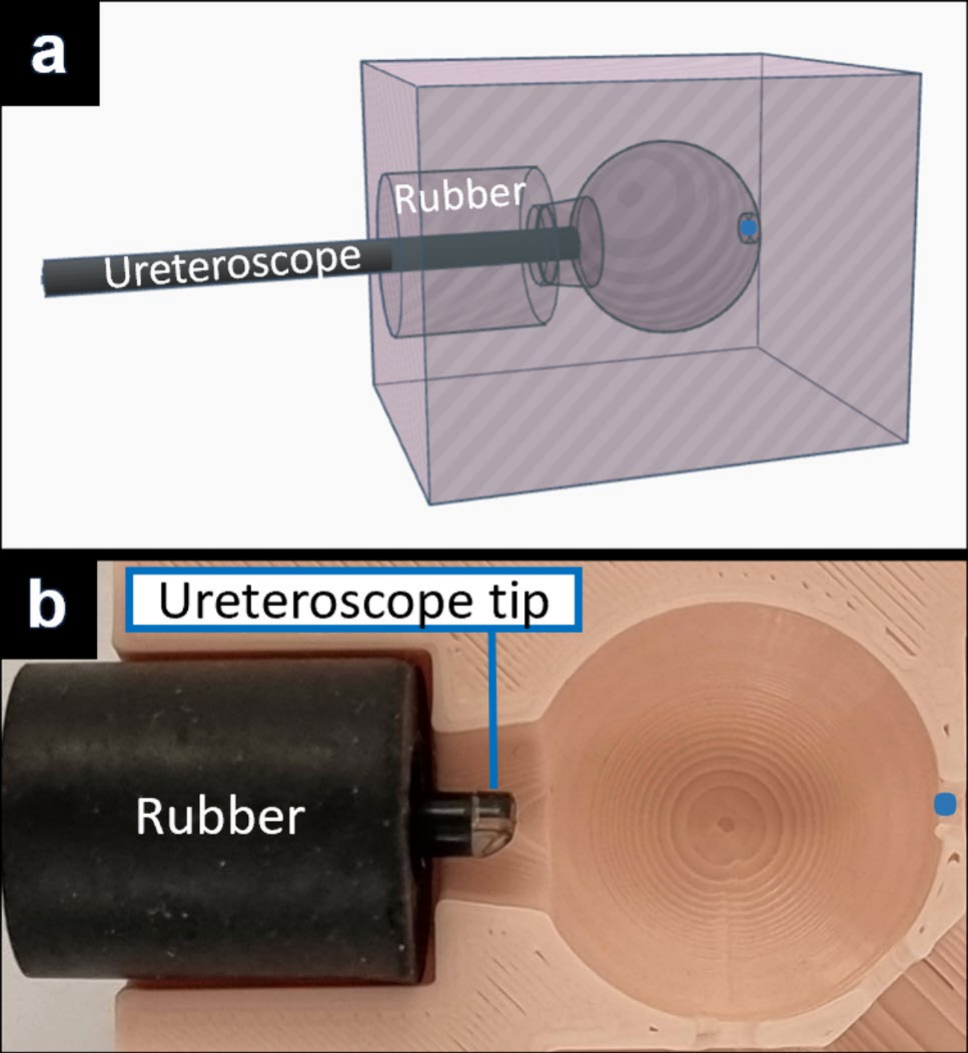
**Experimental setup** **a** 3D modelling of the experimental setup with a ureteroscope inserted into a pink kidney calyx model with a spherical cavity. **b** Cross section of the actual 3D-printed pink model cut in half to display the position of the ureteroscope in relation to the spherical cavity. The black rubber cylinder was used to ensure solid tightening of the ureteroscopes within the model during measurements. The tip of the OTU WiScope is visible and looking out 4 mm from the black rubber for a 20 mm distance between tip of the scope and the center of the concave cavity surface (marked with a blue dot on this figure). The scope was inserted further for capturing images at 5, 10 mm distances (not shown in this figure)

A 3D-printed pink kidney calyx model was used to hold the tested ureteroscope at fixed distances from the concave surface of the kidney calyx model in a dark room (Fig. 1). The model consisted of a closed spherical cavity to replicate the human kidney calyx. Pink was chosen for the kidney calyx model to simulate the color of human urothelial mucosa. The size of the target field and distance from the light sensor was determined with reference to dimensions of models constructed on data from endocasts [24] used in studies investigating laser lithotripsy [25, 26], to reflect in vivo settings. The ureteroscope was maintained in a non-deflected position, with the center of the scope view aligned to the center of the opposite concave pink surface. Endoscopic images were captured with the tip of the ureteroscope at 5 mm, 10 mm and 20 mm distances from the inner surface of the sphere - mimicking situations frequently found in clinical routine. All experiments were performed in saline (NaCl 0.9%) to replicate the usual operative medium in ureteroscopy.

The complete range of brightness settings and brightness modes were evaluated for each scope - where available. These included: First, video image brightness setting (Storz Flex-Xc and Flex-X2s). Second, light stack or processing unit brightness setting (all scopes, except the Storz Flex-Xc). Lastly, brightness mode - Manual vs Auto (Storz Flex-X2s, Olympus V3 and P7). Depending on the scope, some of these adjustment options were not present. This is further described:

For the Storz Flex-Xc, brightness adjustment was available only via the video image brightness menu buttons on the scope handle, and not on the light stack. This was tested at settings of 1/3/5 out of 5. This brightness adjustment function was found not to affect the actual ureteroscope tip light source brightness in previous studies, but only of the projected video image [5, 27, 28]. Similarly, the Storz Flex-X2s had buttons on the camera head allowing for separate brightness adjustment of the video image (tested at settings 1/3/5 out of 5). Assessment of the Flex-Xc and Flex-X2s was done in “Standard” mode with no additional post processing provided by the IMAGE1 STORZ Professional Image Enhancement System (e.g. CLARA/CHROMA).

For all scopes except the Storz Flex-Xc, brightness settings could be changed on the light stack or processing unit, and was tested at the lowest, middle and highest of the available range in each brightness mode and video image brightness setting.

Additionally, the Storz Flex-X2s, Olympus V3 and P7 allowed both automatic and manual modes on the light stack. For the Storz Flex-X2s in manual mode, testing was done with the light stack brightness at 0/50/100% settings. With the automatic mode, the Storz Flex-X2s video image menu interestingly allowed only for setting a specific automatic mode maximum (max) brightness value (50–100%), which when adjusted, was also concurrently reflected on the light stack brightness bar setting. After the ureteroscope was inserted into the model, the light brightness bar on the light stack then adjusts to an “automatic” brightness setting within that maximum value. Images were captured for max 50%/max 75%/max 100% automatic settings on the video image menu. In contrast, for the Olympus V3 and P7 brightness automatic modes, the light stack interestingly allowed setting a fixed brightness value (0–100%), with no subsequent auto adjustment reflected by the light stack brightness bar. The two Olympus scopes were tested at 0/50/100% light stack brightness settings for both auto and manual modes.

For the Pusen 7.5F and 9.2F scopes, testing was done at processing unit light brightness settings of 0/4/7 out of 7, as provided by the manufacturer. For the WiScope, this was tested at 0/50/100% brightness setting, as provided by the manufacturer.

For each different combination of light brightness setting, video capture mode, and distance, a set of five repeated images were captured on the ureteroscope processing unit. Each individual combination is referred to as “situation” from here on. The ureteroscope was withdrawn out of the model and reinserted before each repeated image capture.

### Statistical analysis

All captured images were transferred and analyzed for their histogram grayscale pixel count distribution of brightness using the software *ImageJ* (Version 1.53t RRID: SCR_003070) [29]. This resulted in 256 pixel count measurements for each of the 256 individual histogram grayscale values (histogram scale 0–255). 0 on the scale represents the darkest on the grayscale, and 255 the brightest.

For each situation, the mean pixel count for each 256 individual histogram grayscale values (0, 1, 2, 255) was calculated from the five values of the five repeated captured images, giving a mean histogram grayscale distribution for each individual situation.

**Fig. 2:**
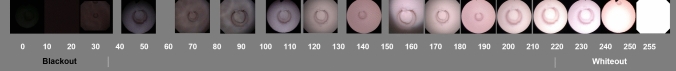
Distribution of endoscopic images over grayscale histograms. Images shown are representative images within the set of five endoscopic images captured for various scopes, at various brightness and distances from the target, arranged in order of the overall histogram median grayscale value for that set of five images. This is meant to illustrate the appearance of blackout and whiteout on the histogram scale (range 0–255). Blackout was defined as 0–35 and whiteout 220–255 on the histogram scale

The overall histogram median value for each situation was defined as the median grayscale value based on the above calculated pixel counts (Fig. 2). All analyses were performed with GraphPad Prism 10.0.1 (GraphPad Software, La Jolla CA, USA).

**Fig. 3:**
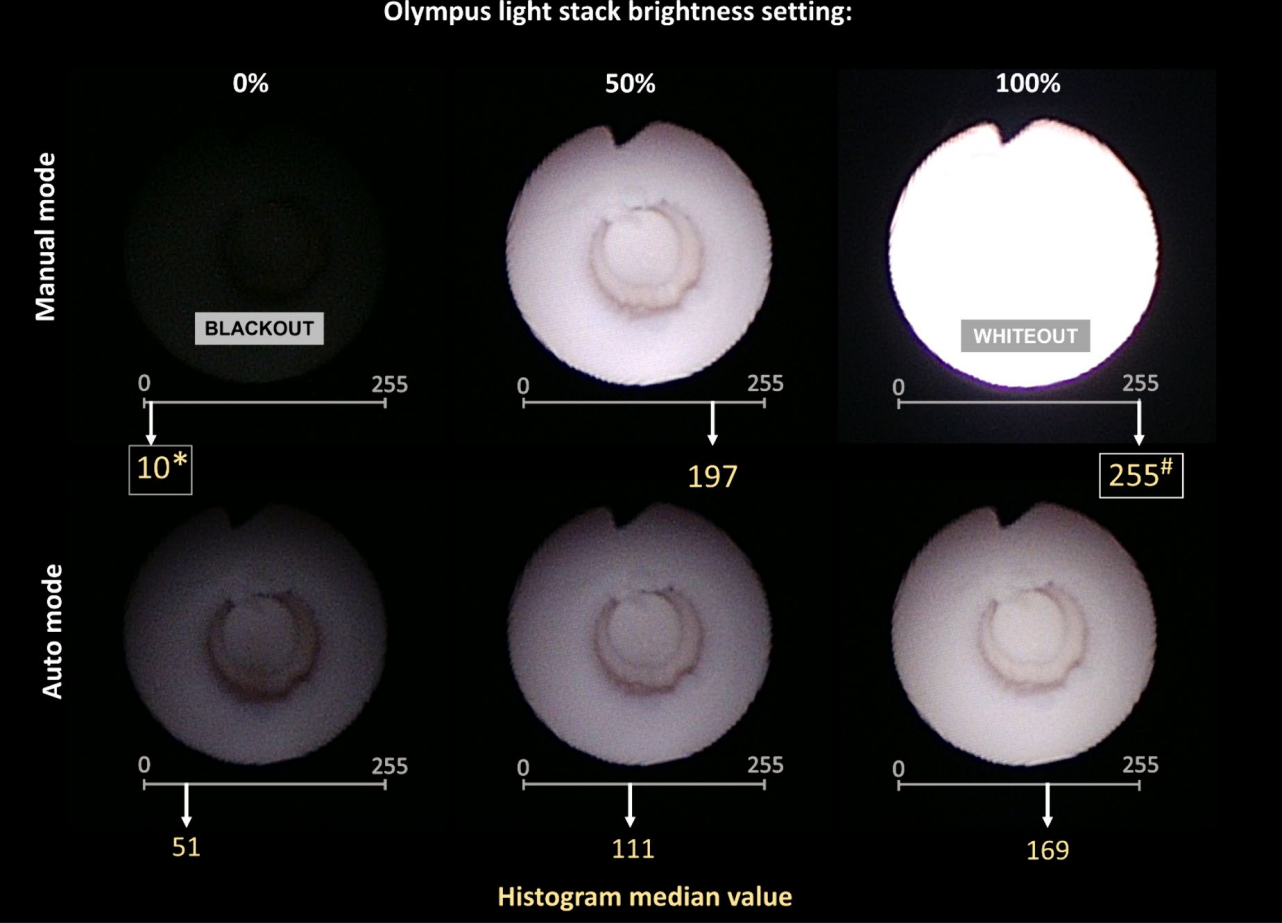
Blackout and whiteout in flexible ureteroscopy. Example images are from the Olympus P7 at 5 mm distance. These are representative images within the set of five endoscopic images captured for each situation. The median histogram value is based on a set of five endoscopic images. The histogram median values are for each brightness setting and mode on the histogram scale of 0 to 255. *Histogram value meeting blackout criteria. ^#^ Histogram value meeting whiteout criteria

Blackout was defined as median histogram range from 0 to 35, and whiteout from range of 220 to 255 (image on monitor too dark or too bright for the human eye, respectively). Cut-offs values were determined by consensus between two authors, J-LK and EXK after review of all captured images. An example of images on a grayscale can be found in Fig. 2, and an example of endoscopic image median histogram values at various brightness settings and modes can be found in Fig. 3.

## Results

### Blackout

Blackout occurred with the P7, Pusen 7.5F and 9.2F, WiScope and at all evaluated distance settings (5 mm, 10 mm, 20 mm) - all with lowest brightness settings. For the V3, this was only found at the furthest 20 mm distance (Table 1).

**Table 1:**
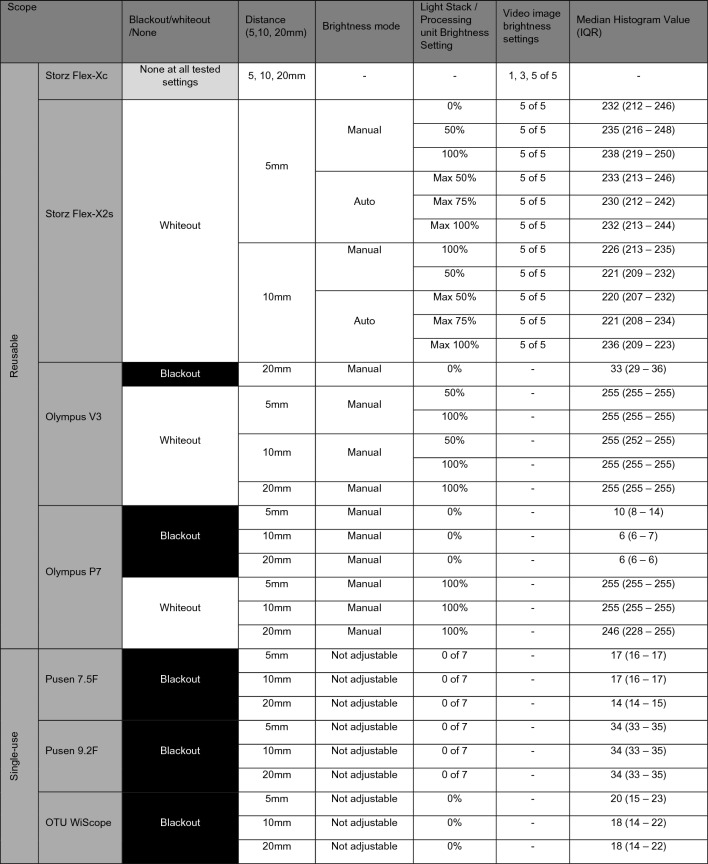
Blackout and whiteout characteristics of flexible ureteroscopes at various distances from target

Settings explored: (1) lowest, mid and highest brightness settings of the available light stack or processing unit for all ureteroscopes, (2) Auto/manual brightness mode for the Storz Flex-X2s, Olympus V3, Olympus P7, (3) Video image brightness setting for Storz Flex-Xc and Flex-X2s

For scopes with adjustable brightness modes, blackout occurred only in the manual mode and not in automatic mode (V3 and P7). The Flex-X2s did not have blackout even in manual mode.

Interestingly, the Flex-Xc and Flex-X2s had no blackout even at the furthest 20 mm distance and lowest brightness mode setting available.

### Whiteout

Whiteout occurred with the Flex-X2s, V3 and P7 at 5 mm and 10 mm distance, as well as at 20 mm distance with the V3 and P7 - mostly with 50–100% brightness settings (Table 1).

For scopes with adjustable brightness mode, whiteout happened for the Flex-X2s in both manual and automatic mode, mostly when the video image capture setting was five out of five. For the V3 and P7, whiteout happened exclusively in the manual mode at all tested distances (5, 10, 20 mm).

### No blackout or whiteout

The Flex-Xc was the only scope that had neither blackout nor whiteout over all brightness settings and brightness modes.

## Discussion

Our findings reveal that within the range of brightness settings available, some scopes can blackout or whiteout, while others do not. This phenomenon mainly occurred when light source brightness or image brightness settings were at their extreme low or high ends. These undesirable settings may occur during clinical routine, whether consciously or inadvertently set by operating room staff or members.

Blackout of scopes is particularly undesirable, as the surgeon would like to maintain a decent level of vision even at the lowest brightness setting of the scope. The only rare situation where blackout is desired is in a setting of an endoscope meeting procedure, typically referred to as a “cut to the light” procedure [30]. In this special situation, one would need a scope that is capable of switching off its light source to artificially create a blackout situation. From there, the light source of the second endoscope can be searched for. All single-use scopes in our study had blackout at all distances with the lowest brightness settings. It would be of interest to test more single-use scopes to see if this characteristic is found in other single-use scopes.

Generally, whiteout happens during higher light stack brightness settings for the Flex-X2s, V3 and P7. This happens as well during highest settings on the Flex-X2s video image brightness settings. Based on these findings, it may be prudent for most scopes to start at 50% brightness light stack brightness setting and 3 out of 5 video image brightness setting (when available), before adjusting for the particular in vivo situation. It is also important to be aware that some scopes like the Flex-X2s, depending on the camera head used, may have both light brightness settings on the light stack and on the camera head video image menu.

The automatic mode of the modern ureteroscopic light stack helps to adjust the brightness of the image. Based on our findings of whiteout happening mostly in the manual mode for scopes with adjustable modes, the authors would recommend keeping light mode on automatic for most clinical applications. It is also interesting that we were able to adjust brightness settings in the auto mode for the Flex-X2s, V3 and P7, influencing brightness in an “automatic” mode.

The present study is not devoid of limitations. First, the authors arbitrarily used median histogram value cut-offs to determine blackout and whiteout criteria. The strength of this methodology is that it is based on histogram values which provide an objective way to qualitatively determine blackout and whiteout in images from scopes. Second, this is an in-vitro attempt to reflect in vivo use of ureteroscopes. Still images were used instead of videos, with the methodology dictating an objective assessment via still image histogram values. If videos were used, this would be technically challenging to compute for each frame of the video. To ensure reliability of the results, a set of five images was captured for each individual combination of brightness setting, brightness mode and distance. Environmental factors may play a role in affecting the clinical translation of our study findings, and thus interpretation of the data must, therefore, be taken with care. The effect of blood clots, stone dust and urine on blackout and whiteout characteristics will need to be further evaluated. Third, other situations not evaluated in this study include that of situations in the ureter with the enclosed working cavity being a hollow tube rather than spherical in nature. This warrants further evaluation.

## Conclusions

Blackout or whiteout of images is an undesirable property that was found for several scopes, possibly impacting diagnostic and therapeutic purposes during ureteroscopy. These observations form a guide for urologists which may impact their choice of instruments and corresponding settings.

## Data Availability

On request to corresponding author for raw data on the experimental setup.
